# Combination resonance and primary resonance characteristics of a dual-rotor system under the condition of the synchronous impact of the inter-shaft bearing

**DOI:** 10.1038/s41598-023-27922-8

**Published:** 2023-01-20

**Authors:** Guanshu Zuo, Lei Hou, Rongzhou Lin, Shuangxing Ren, Yushu Chen

**Affiliations:** School of Astronautics, Harbin Institute of Technology, Harbin, 150001 China

**Keywords:** Aerospace engineering, Mechanical engineering

## Abstract

The internal structure of many aero-engines is designed with a dual-rotor system. Up to now, there have been few studies on the influence of aerodynamic excitation on the dual-rotor system. The phenomenon of synchronous impact may occur when the frequency of the aerodynamic excitation force of the fan blade is close to the characteristic frequency of the inter-shaft bearing. This paper investigates the dynamic characteristics of a dual-rotor system under the condition of synchronous impact. The system's motion equations are formulated considering the complex nonlinearities of the inter-shaft bearing, such as Hertz contact force of 10/9 exponential function, clearance, and periodic varying compliance. In addition, the inter-shaft bearing with a local defect is considered. The fan blade’s aerodynamic excitation force is modeled by synthesizing multiple harmonic excitation forces, the amplitudes of which are obtained by the Fourier series expansion. Numerical simulations are employed to get the dynamic responses of the system. The results show that the dynamic characteristic of the dual-rotor system at the primary resonance caused by the high-pressure (H.P.) rotor is not changed by the aerodynamic excitation force, while the primary resonance caused by the low-pressure (L.P.) rotor increases significantly. However, three aerodynamic resonances of the amplitude-frequency response of the dual-rotor system are emerging in the low-frequency region (124, 146 and 186 rad/s). When the synchronous impact phenomenon occurs, the amplitude of the three resonance peaks will increase twice compared to the original status, leading to a doubled increase in the dynamic load of the inter-shaft bearing. The characteristics of the dual-rotor system affected by the parameters such as initial phase difference of local defect, rotor eccentricity of system, clearance of inter-shaft bearing, and the stiffness and damping of supports are discussed in detail. The results obtained provide a deep insight into the mechanism of synchronous impact.

## Introduction

With the development of lightweight and high-speed aero-engine in recent years, the rotor blades are subjected to more airflow force and centrifugal force, resulting in more frequent vibration failures of engine blades. Almost all aero-engines have experienced blade vibration failures caused by the excitation of airflow in production, experiment, and operation, especially in the research and development stage of new engines. Such losses are relatively common^1,2^. Many theoretical analyses and experimental research have been carried out to solve this problem. Dafaye et al.^3^ demonstrated that integrating a fluid layer on the inter-shaft bearing makes it feasible to reduce the shocks on each rotor, and their impact on the dynamic characteristics of a dual-rotor engine was analyzed to meet the goal of decreasing the embarking mass. Mailach et al.^4^ pointed out that the upstream rotor blade's wake and the downstream rotor blade’s potential interference flow greatly influence the distribution of the pressure on the stator blade surface. They demonstrated that the frequency of excitation force on the blade surface is the fundamental frequency and its multiple frequencies of the rotation frequency of the upstream blade. Leinhos et al.^5^ explored instabilities in the initial phase and the early identification of stall precursors by using the signals of high-frequency pressure at various engine power levels. At the same time, the compressor displayed several forms of stall inception based on engine frequency. Moore^6^ demonstrated how the temporary stall mechanism modifies the instantaneous compressor pumping profile. Ishii et al.^7^ paid attention to the numerical model for multiblade-row compressors to obtain the influence of multiple factors on the surge and rotating stall inception and post-stall dynamics in multistage axial compressors. Hoss et al.^8^ observed technological tools, including periodic low-pass and band-pass filtering, spatial and temporal Fourier transforms, and a wavelet transform, to detect three different types of L.P. compressor stall inception processes. Wilson et al.^9^ detailed the progression of flow instability at varying speeds, out of a small stopped fluid zone, including simply just a few blades to the entire machine’s dramatic surge action. Weston et al.^10^ worked on a series of dynamics simulations. They demonstrated that as the fan got closer to stall and the degrees of overall temperature distortion rise, the rotation of the distortion became more evident. Yamada et al.^11^ discussed the flow mechanism of the rotating stall inception; many simulations show that the spinning stall spreads to the upstream and downstream blade rows because of its significant blocking impact. Giuliani et al.^12^ presented flow solutions, verified the experimental data to validate the solver modifications, and inspected flow pathways around the blades to further understand the stalled process. Soderquist et al.^13^ explained the connections between the variations in circumferential rotor power and the inlet total pressure distortion, pressure-induced swirl, total pressure distortion transfer, and total temperature distortion production. An et al.^14^ used numerical analysis to examine the complicated flow fields in a transonic axial flow compressor rotor's blade tip area, and the characteristic frequency and amplitude of the flow oscillation showed a significant shift with the modification of the vortex breakdown shape.

Particular progress has also been made in the research on dual-rotor systems. Tiwari et al.^15,16^ and Harsha et al.^17–19^ studied the nonlinear dynamic reactions of balanced and unbalanced stiff rotors supported by rolling bearings, varying speeds and different radial internal clearances were considered. The results showed that the rotor speed and radial internal clearance considerably influence the formation of periodic, subharmonic, and chaotic behaviour. Ferraris et al.^20^ provided an analytical expression of unbalanced mass response and the critical speed on a dual-rotor system model, where the nonlinear dynamic characteristics of asymmetric coaxial corotating and counter-rotating rotor systems are discussed. Hu et al.^21^ developed a complex nonlinear typical model with 5-DOF for a dual-rotor system by considering bearing radial clearance. The simulation results showed that the rotational flexibility of rotors greatly influences the system’s dynamic simulation. Hou et al.^22^ applied HB-AFT to solve a complex nonlinear model of a dual-rotor inter-shaft bearing system with four degrees of freedom. The effect of speed ratio, unbalanced excitation and inter-shaft bearing's clearance in the radial direction on the system's amplitude-frequency characteristics was demonstrated. Liu et al.^23^ suggested a dynamic model with two different pressure rotors; the critical speed, vibration frequency, and LP rotor's vibration responses were investigated. Lu et al.^24^ investigated the nonlinear properties of a dual-rotor system connected with an inter-shaft bearing and unbalanced excitations, vertical static forces, and gravities to establish the system's motion equations. Gao et al.^25,26^ paid attention to the jump and hysteresis phenomenon by considering the inter-shaft bearing as a pressure plate spring whose characteristic was approximately nonlinear. Jin et al.^27^ used the FE method to construct the model of a dual-rotor inter-shaft bearing system with two levels of the order reduction method. Zhao et al.^28^ investigated the dynamic characteristic of dual-rotor systems with nonlinear rubbing faults and multi-frequency excitations. It demonstrated that the dual-rotor system with nonlinear rubbing fault would produce abundant combination frequency components. Fu et al.^29^ presented a dual-rotor system supported by ball bearings’ nonlinear vibration responses with coupling misalignment taken into account. Several physical parameters were examined to show how their uncertainty affects the nonlinear vibrations at various rotational speeds. Lu et al.^30^ concentrated on a dual-rotor system supported by a rolling element bearing, the ball bearing’s nonlinear properties like time-varying stiffness and primary resonance were concerned. The results showed that when the ball bearing's clearance raised, the system's stiffness and the rotation speed for forced resonance decreased.

Researchers mainly adopted the centralized parameter modeling method to study the problem of simulating the vibration characteristics of bearings induced by local defects. Sun et al.^31^ investigated the dual-rotor system’s responses and stability, obtained the complicated nonlinear phenomena, and offered further insight into the response properties of the dual-rotor system with rub-impact. Gao et al.^32^ also studied a local defect on the outer race and focused on the effects of dynamic characteristics, four anomalous resonances were remarked in the curve of frequency response which was caused by the local defect. Patil et al.^33^ established a bearing analysis model based on the Hertz model to simulate the vibration characteristics of a bearing with a single defect. They studied the influence of the defect’s size and location on the amplitude of the acceleration. Sawalhi et al.^34^ proposed a 5-DOF bearing pedestal model by using Hertz model, which can simulate the local defects of bearing, and combined with a simplified gear model to establish a model of the gearbox test bench. Khanam et al.^35^ put forward a dynamic model of bearings excited by multiple excitations, defined the exiting process as an impact excitation, and analyzed in detail the motion mechanism of rollers when passing through the front and rear edges of defects. Cui et al.^36^ compared and analyzed the simulation signal and experimental data of the 4-DOF model and explored the quantitative diagnosis method of a single defect in the outer ring by measuring the time interval between two impacts of roller going in and out faults. Patel et al.^37^ put forward the dynamic model of deep groove ball bearing and studied the vibration response of the bearing’s inner and outer race with single-point and multi-point defects.

It can be found that almost all the research above focused on the rotor blades' vibration or the nonlinear vibration of the rotating rotor. However, Wang et al.^38^ pointed out that a synchronous impact phenomenon may occur when the frequency of the aerodynamic excitation force and that of the roller impact force were in the same frequency and phase. The phenomenon may increase the dynamic stress on the inter-shaft bearing, which cannot be conducive to safe and stable operation. Therefore, it is essential to reveal the mechanism of the synchronous impact of the system subjected to aerodynamic excitation forces.

There has been no research on the mechanism of synchronous impact for a dual-rotor or a three-rotor system. The vibration characteristics caused by synchronous impact are also unclear. Studying the resonance characteristics of a dual-rotor system under the synchronous impact is necessary, which will be helpful for the synchronous impact fault diagnosis.

The motivation of this paper is to detect the dynamic characteristics of the dual-rotor system under the synchronous impact of the inter-shaft bearing. The motion equations of the dual-rotor system are formulated with the impact force of the inter-shaft bearing and the aerodynamic excitation force, wherein the local imperfection on the surface of the inter-shaft bearing's inner race is considered. The nonlinearities such as Hertz contact force of 10/9 exponential function, clearance and varying compliance are included. Moreover, the aerodynamic excitation force is modelled by synthesizing multiple harmonic excitation forces, the amplitudes obtained by the Fourier series expansion. This model enables us to conduct a deep systematic investigation of the nonlinear dynamic characteristics of the dual-rotor and the emotional load of the inter-shaft bearing. The dynamic characteristics of the system affected by the parameters (initial phase difference of local defect, rotor eccentricity of system, clearance of inter-shaft bearing, the stiffness and the damping of supports) are discussed in detail to provide a profound insight into the mechanism of the synchronous impact of the dual-rotor system.

This paper is divided into four parts. "Introduction" Section introduces the background and the research status. "Dynamic modelling of the dual-rotor system" Section establishes the model of a dual-rotor inter-shaft bearing system with a local defect. The motion equation of the system is obtained through the second Lagrange equation. In "Numerical results and discussions" Section, the characteristics of the dual-rotor system affected by the parameter are discussed in detail by the Runge–Kutta method. At the end of this article, the conclusions are summarized in Section "Conclusions".

## Dynamic modelling of the dual-rotor system

### Dual-rotor system with a local defect on the surface of the inter-shaft bearing

The frequency of the aerodynamic excitation force is the product of the number of fan blades and the speed of the low-pressure rotor, so it is a high-frequency excitation source, which was not considered in the dual-rotor system before. This novel excitation source mainly acts on the fan blade of the LP rotor and is transmitted to the inter-shaft bearing through the shaft of the LP rotor, finally coupled with the roller impact force of the inter-shaft bearing. The synchronous impact will occur when the frequency of the bearing roller impact force is consistent with that of pneumatic exciting force.

**Figure 1:**
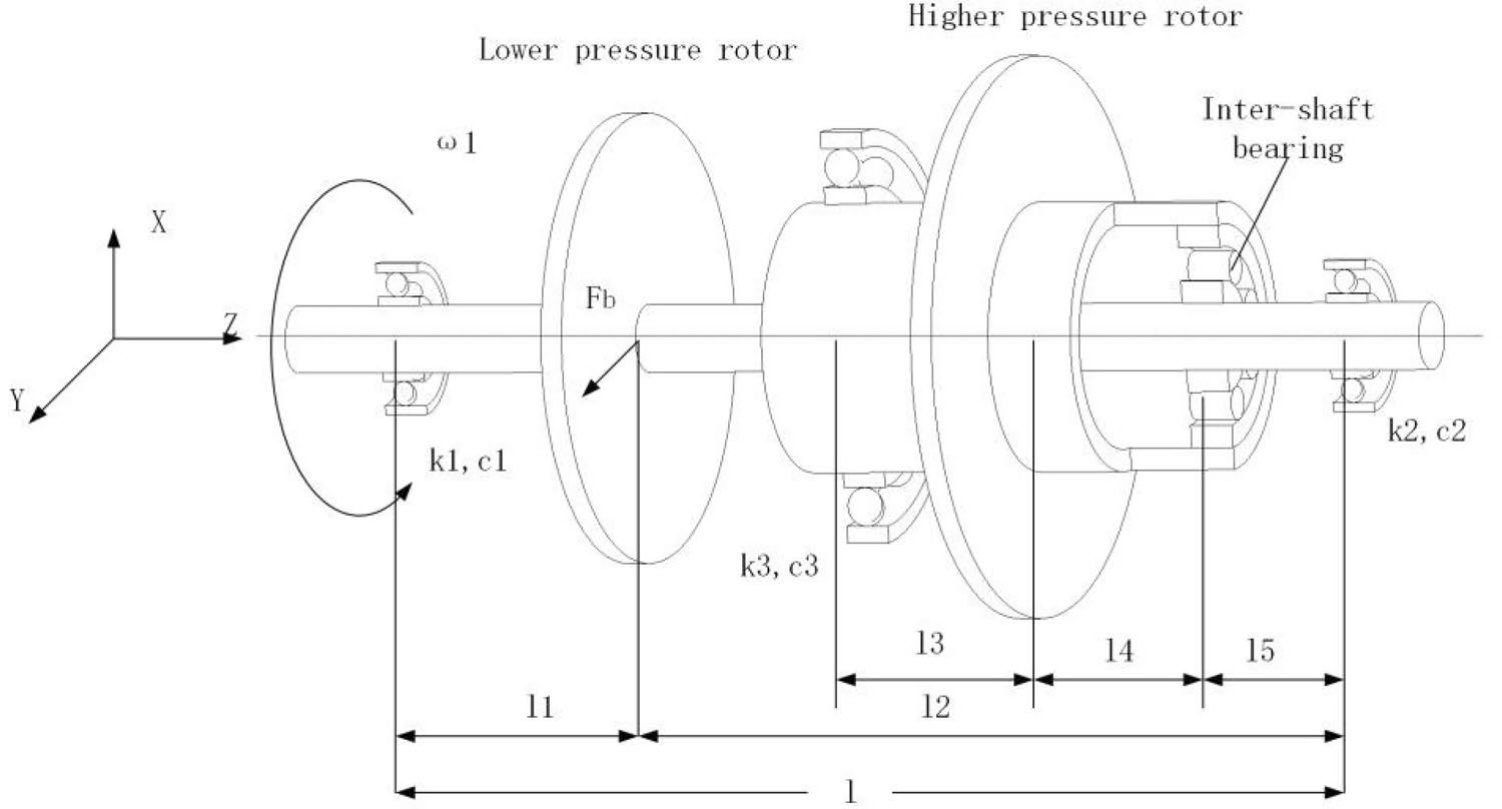
Schematic diagram of a typical dual-rotor system.

In this paper, the modeling is formulated based on a rigid rotor, for the synchronous impact mainly occurs in the low-frequency region, and the deformation of the rotor is minimal when the rotor speed is low. There is a long distance from the primary resonance of the system, thus the dual-rotor system in this state can be approximately considered a rigid rotor. A typical dual-rotor model is shown in Fig. 1, which contains two different rotors. The LP rotor is supported by two precise fixed deep groove ball bearings. The HP rotor is held up by an accurate fixed deep groove ball bearing and a cylindrical roller bearing named inter-shaft bearing, connecting the HP rotor and LP rotor. The turbines and the compressors of the HP rotor and LP rotor are simplified into two thin discs with misaligned centroids^32^. Since the rigidity coefficients of the supports are relatively large, the forces are approximately linear, while the nonlinear Hertz contact force is considered. Moreover, the aerodynamic excitation force from the fan blade is considered, which is loaded on the LP rotor.

The kinetic energy of the system is denoted as

1a $$T_{1} = \frac{1}{2}m_{1} (\dot{x}_{1}^{2} + \dot{y}_{1}^{2} ) + \frac{1}{2}J_{d1} (\dot{\theta }_{x} + \dot{\theta }_{y} ) + \frac{1}{2}J_{p1} (\omega_{1}^{2} - 2\omega_{1} \theta_{x} \dot{\theta }_{y} ),$$

1b $$T_{2} = \frac{1}{2}m_{2} (\dot{x}_{2}^{2} + \dot{y}_{2}^{2} ) + \frac{1}{2}J_{d2} (\dot{\phi }_{x} + \dot{\phi }_{y} ) + \frac{1}{2}J_{p2} (\omega_{2}^{2} - 2\omega_{2} \phi_{x} \dot{\phi }_{y} ),$$

The potential energy of the system is denoted as

2a $$V_{1} = \frac{1}{2}k_{1} [(x_{1} - \theta_{y} l_{1} )^{2} + (y_{1} + \theta_{x} l_{1} )^{2} ]$$

2b $$V_{2} = \frac{1}{2}k_{2} [(x_{1} + \theta_{y} l_{2} )^{2} + (y_{1} - \theta_{x} l_{2} )^{2} ],$$

2c $$V_{3} = \frac{1}{2}k_{3} [(x_{2} - \phi_{y} l_{3} )^{2} + (y_{2} + \phi_{x} l_{3} )^{2} ]$$

The dissipated energy of the system is denoted as

3a $$D_{1} = \frac{1}{2}c_{1} [(\dot{x}_{1} - \dot{\theta }_{y} l_{1} )^{2} + (\dot{y}_{1} + \dot{\theta }_{x} l_{1} )^{2} ],$$

3b $$D_{2} = \frac{1}{2}c_{2} [(\dot{x}_{1} + \dot{\theta }_{y} l_{2} )^{2} + (\dot{y}_{1} - \dot{\theta }_{x} l_{2} )^{2} ],$$

3c $$D_{3} = \frac{1}{2}c_{3} [(\dot{x}_{2} - \dot{\phi }_{y} l_{3} )^{2} + (\dot{y}_{2} + \dot{\phi }_{x} l_{3} )^{2} ],$$

Subscript 1 denotes the LP rotor, whereas subscript 2 denotes the HP rotor in the equations above; $J_{d}$, $J_{p}$, $\omega$, $e$, $m$ indicate the rotor’s diameter moment of inertia, the rotor's polar moment of inertia, the disk's rotation speed, the rotor's eccentricity and the rotor disk’s mass; $x$, $y$ indicate the displacements of disks in the vertical and horizontal direction. $\theta_{x}$ and $\theta_{y}$ display the rotation angle of the LP disk around the x and y directions.$\varphi_{x}$, $\varphi_{y}$ represent the HP disk's rotation angle around x and y directions; $c_{i}$ and $k_{i}$ ($i = 1\sim 3$) indicate the supports' damping and stiffness coefficient, respectively. $F_{x}$, $F_{y}$ denote the bearing's force in the vertical and horizontal orientation.

### Force of the inter-shaft bearing with defect

The process of the rollers going into the local defect and leaving the local defect can be simulated by establishing a model of inter-shaft bearing with a defect on the inner race surface, the defect depth is shallow and the span is significant. The defect has little influence on the rigidity of the rollers and the bearing rings but significantly affects the deformation of the bearing rollers. The rigidity of the bearing with a local defect is relatively large, so the bearing can be regarded as an elastic material. When the rollers of the bearing pass through the defect, the sliding friction of the bearing does not need to be considered. The contact area of the roller in the defect is small compared with the radius of the bearing. Therefore, the Hertz basic assumptions^39^ of the bearing are changeless during the rollers going in and out of the defect, meanwhile the Hertz model can be formulated to calculate the bearing restoring force with a local defect. Compared with the manifestation and mechanism of roller impact force, the frequency of roller impact force has a more significant influence on the dual-rotor system, the frequency is coupled with that of aerodynamic exciting force to produce a synchronous impact phenomenon. Therefore, the defect frequency caused by the roller impact force of the bearing can be well reflected by introducing the geometric defect model, and the process of the rollers passing through the defect can be simulated.

**Figure 2:**
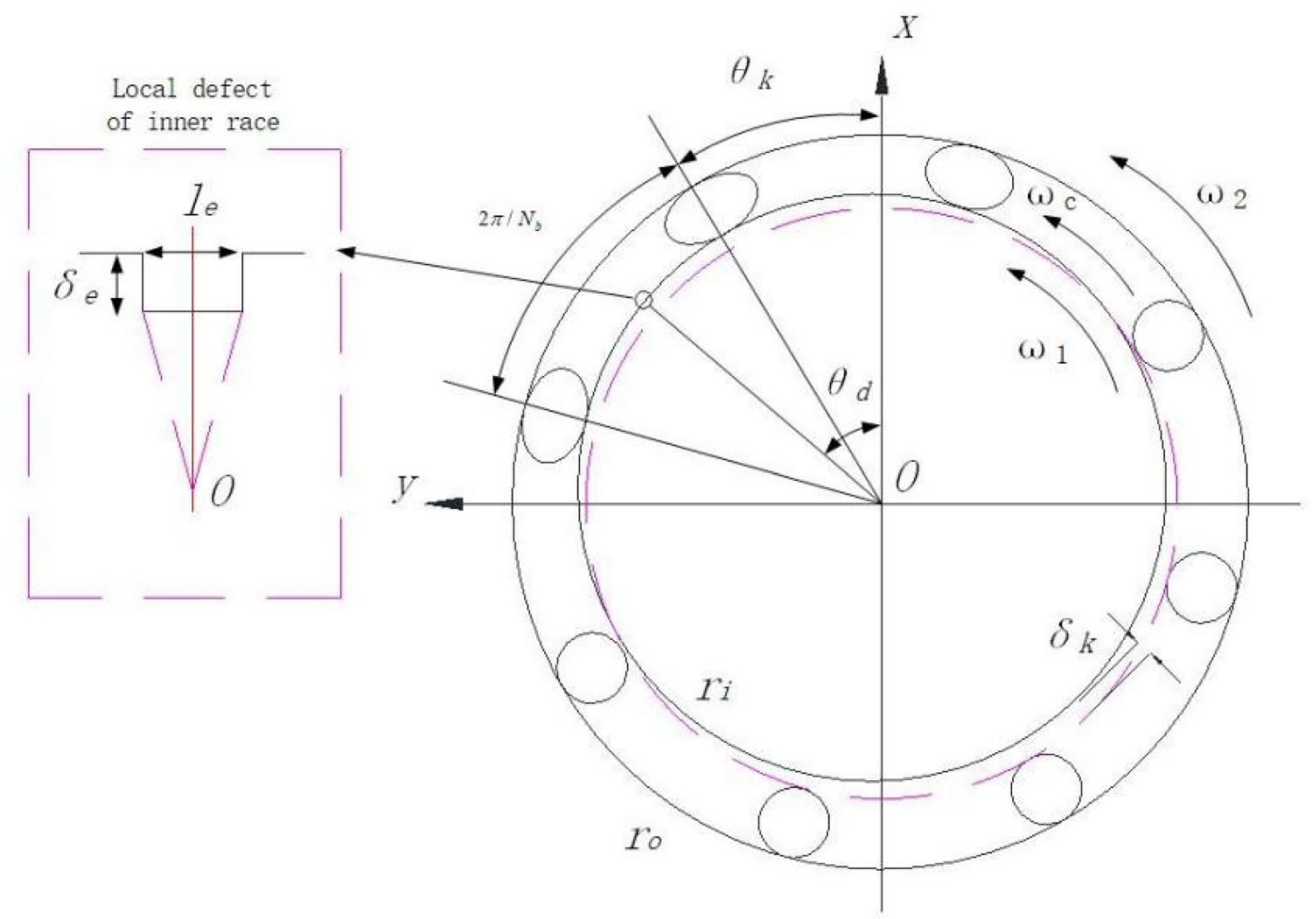
Schematic diagram of the inter-shaft bearing.

Based on the above reasons, the Hertz restoring force modelling can be considered in the model to solve the vibration characteristics of the bearing^40–42^. Figure 2 shows a simplified schematic representation of the inter-shaft bearing, *ɷ*_1_ is the rotation speed of the inner race, *ɷ*_2_ is the rotation speed of the outer race, *ɷ*_*c*_ is the rotation speed of the cage, *δ*_*e*_ is the depth of the defect, and *l*_*e*_ is the defect’s span.

Since the inter-shaft bearing’s inner and outer races are continually spinning, the instantaneous angular position of local defects can be expressed by time *t*, and the parameter *θ*_*e*_ is the instantaneous angle of local defect, initial angle position *φ* of local defect at any time *t* can be expressed as

4 $$\theta_{e} = \omega_{1} t + \phi .$$

The deformation caused by the restoring force between the *k*th roller and bearing ring is

5 $$\delta_{{k_{d} }} = \left\{ \begin{gathered} \delta_{k} - \delta_{e} \left( {\Delta \theta_{k} < \frac{1}{2}\frac{{l_{0} }}{{r_{i} }}} \right) \hfill \\ \delta_{k} \left( {\Delta \theta_{k} \ge \frac{1}{2}\frac{{l_{e} }}{{r_{i} }}} \right) \hfill \\ \end{gathered} \right.$$

While the restoring force of the *k*th roller is

6 $$F_{k} = K_{b} \delta_{{k_{d} }}^{n} G\left( {\delta_{{k_{d} }} } \right)$$

Then the bearing's restoring force can be expressed as follows

7 $$\left[ \begin{gathered} F_{x} \hfill \\ F_{y} \hfill \\ \end{gathered} \right] = K_{b} \sum\limits_{k = 1}^{{N_{b} }} {(\delta_{kd}^{n} G[\delta_{kd} ])} \left[ \begin{gathered} \cos \theta_{k} \hfill \\ \sin \theta_{k} \hfill \\ \end{gathered} \right],$$

where $$G[\delta_{kd} ] = \left\{ \begin{gathered} 1(\delta_{kd} > 0) \hfill \\ 0(\delta_{kd} \le 0) \hfill \\ \end{gathered} \right.$$, *K*_*b*_ is the inter-shaft bearing's Hertz contact stiffness, and the index n of contact deformation is $\frac{10}{9}$ because the cylindrical roller bearing is usually used as an inter-shaft bearing in the aero engine field.

When the roller rolls past the local damage on the ring, the ring and roller will produce displacements at the corresponding damage, generating a series of impact forces. This impact force is an essential input for the dynamic model of bearing failure, so the impact of the faulty bearing during operation cannot be ignored.

**Figure 3:**
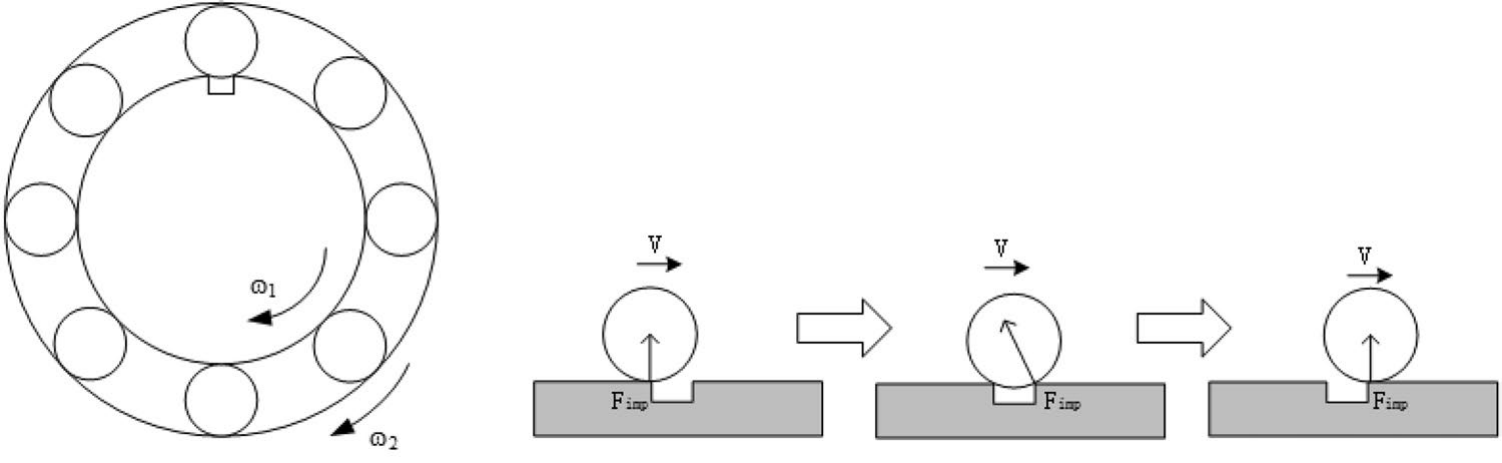
Schematic diagram of bearing roller impact process.

The micro-damage point on the inter-shaft bearing is impacted once whenever it contacts a roller, and the magnitude of the impact force direction is related to the position size and other parameters of the bearing defect point, as indicated in Fig. 3.

According to Fig.. 3, the impact force of the roller on the bearing is not strictly against the geometric centre of the bearing, and the angle between the direction of the impact force and the centre of the bearing circle is $\eta = \arcsin \left( {\frac{{2r_{b} }}{{l_{e} }}} \right)$, where *r*_*b*_ is the roller radius, *l*_*e*_ is the span of the bearing defect. To simplify the model, two cases are considered in the following:

1. Suppose that the bearing defect span *l*_*e*_ is minimal, and the angle of the impact force $\eta$ can be approximated as 0; that is, the direction of the impact force is always towards the geometric centre of the bearing.
2. Suppose the bearing defect span *l*_*e*_ is relative to *r*_*b*_ is relatively large, and it can be approximated that the angle of the impact force $\eta$ is 90°; that is, the direction of the impact force is always towards the tangential direction of the bearing.

**Figure 4:**
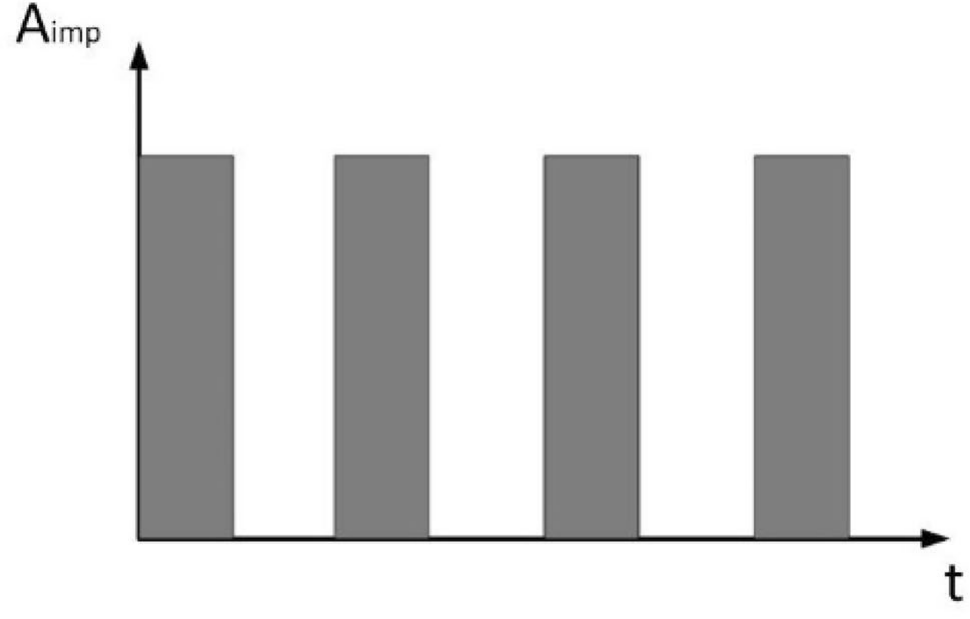
Roller impact force.

By now, three main impact functions express the impact force of a bearing: rectangular, triangular and half-sine functions^43^. In this paper, it is assumed that the impact force of the bearing is constant and is not related to other factors such as relative roller speed and material. The impact force is generated when the roller enters the defect range until the roller leaves the defect range; the roller impact force function is rectangular, which can be expressed as shown in Fig. 4.

In the concepts of bearings, the frequency of the impact force is called the bearing’s passing frequency, which is expressed as follows

8 $$f_{imp} = \frac{{N_{b} \left| {f_{l} - f_{o} } \right|}}{2}\left( {1 + \frac{d}{D}} \right),$$

where $d = r_{o} - r_{i}$, $D = r_{o} + r_{i}$, *N*_*b*_ is the number of rollers, $f_{l}$ and $f_{o}$ indicate the rotation frequency of the LP and HP rotor, $A_{imp} = 200$.

Since the defect is found on the surface of the bearing's inner race, the impact force of the roller can be expressed as

9 $$\left[ {\begin{array}{*{20}c} {F_{impx} } \\ {F_{impy} } \\ \end{array} } \right] = F_{imp} (t)\left[ {\begin{array}{*{20}c} {\cos (\omega_{1} t + \psi )} \\ {\sin (\omega_{1} t + \psi )} \\ \end{array} } \right],$$

where *ψ* is the initial angle of the defect position of the inner race.

### Aerodynamic excitation force

**Figure 5:**
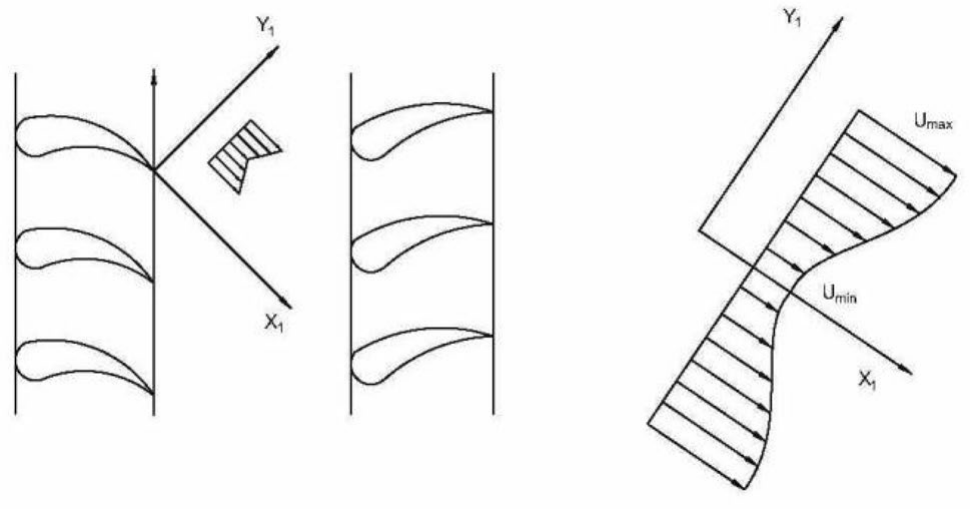
Schematic diagram of aerodynamic excitation force of inlet guide blade.

The aerodynamic excitation force mostly appears in the guide vane-rotor structure (wake excitation) of water pumps, steam turbines and large centrifugal compressors, mainly due to the obstruction of the design for the flow of wake. In the domain of the axial flow compressor, the influence of the blade trailing edge and boundary layer will cause the loss of cascade outlet flow speed, as shown in Fig. 5, because the downstream cascade has a relative rate with the upstream cascade, the aerodynamic parameters at the inlet of the downstream cascade change periodically so that the rotor blades will be subjected to a periodic excitation. The excitation of the flow on the rotor blade is an essential factor of the dynamic stress, which is directly related to the life and safety of the blade.

The fan blade's aerodynamic excitation force is modelled by synthesizing multiple harmonic excitation forces, the amplitudes of which are obtained by the Fourier series expansion. When the frequency of the resonance force is close to or equal to a particular order of the natural frequency of the rotor blade, it will arouse resonance. The airflow parameters are uniform along the circumferential direction for the blade cascade with uniformly distributed blades, except for the blade wake. Each rotation of the rotor is only excited by *Z*_*b*_ wake excitations, *Z*_*b*_ is the number of guide blades.

The aerodynamic excitation force of a single blade can be expressed as $P_{i} = P\sin (2\pi ft + i\varphi_{0} )$^44^, where *i* is the number of blades and $\varphi$ is the phase difference between the blades of two impellers.

For a rotor with *Z*_*b*_ blades, the Fourier expansion of the aerodynamic excitation force can be written in the following form^24^.

10 $$F_{b} (t) = F_{0} \left[ {1 + \cos \left( {Z_{b} \Omega t + \phi_{1} } \right) + \frac{1}{2}\cos \left( {2Z_{b} \Omega t + \phi_{2} } \right) + \frac{1}{3}\cos \left( {3Z_{b} \Omega t + \phi_{3} } \right)} \right],$$

where *F*_0_ is determined by the factors such as the pitch of the cascade, the clearance between the rotor and stator, and the thickness of the trailing edge. The eccentric direction of the LP rotor is the same as the direction of *F*_*b*_. For the convenience of calculation, it is assumed that *F*_0_ is constant.

Roller impact force and aerodynamic excitation force are considered external forces. In this paper, it is assumed that the defect span is small; that is, the direction of the roller impact force is toward the bearing axis. Since the aerodynamic excitation force acting on the LP rotor is mainly considered in this paper actually, the aerodynamic excitation force can be expressed as follows according to Eq. (10)

11 $$\left[ {\begin{array}{*{20}c} {F_{bx} } \\ {F_{by} } \\ \end{array} } \right] = F_{b} (t)\left[ {\begin{array}{*{20}c} {\cos (\omega_{1} t)} \\ {\sin (\omega_{1} t)} \\ \end{array} } \right].$$

### Dynamic model of the dual-rotor system considering aerodynamic excitation force

According to the second Lagrange equation, considering the impact force of inter-shaft bearing and aerodynamic excitation force, by using the Eqs. (1)–(11), the dynamic equations can be obtained as

12a $$\begin{gathered} m_{1} \ddot{x}_{1} + c_{1} (\dot{x}_{1} - L_{1} \dot{\theta }_{y} ) + c_{2} (\dot{x}_{1} + L_{2} \dot{\theta }_{y} ) + k_{1} (x_{1} - L_{1} \theta_{y} ) \hfill \\ \quad + k_{2} (x_{1} + L_{2} \theta_{y} ) + F_{x} = m_{1} e_{1} \omega_{1}^{2} \cos (\omega_{1} t) - F_{impx} - F_{bx} - m_{1} g, \hfill \\ \end{gathered}$$

12b $$\begin{gathered} m_{1} \ddot{y}_{1} + c_{1} (\dot{y}_{1} + L_{1} \dot{\theta }_{x} ) + c_{2} (\dot{y}_{1} - L_{2} \dot{\theta }_{x} ) + k_{1} (y_{1} + L_{1} \theta_{x} ) \hfill \\ \quad + k_{2} (y_{1} - L_{2} \theta_{x} ) + F_{y} = m_{1} e_{1} \omega_{1}^{2} \sin (\omega_{1} t) - F_{impy} + F_{by} , \hfill \\ \end{gathered}$$

12c $$\begin{gathered} J_{d1} \ddot{\theta }_{x} + J_{p1} \omega_{1} \dot{\theta }_{y} + c_{1} L_{1} (L_{1} \dot{\theta }_{x} + \dot{y}_{1} ) + c_{2} L_{2} (L_{2} \dot{\theta }_{x} - \dot{y}_{1} ) + k_{1} L_{1} (L_{1} \theta_{x} + y_{1} ) \hfill \\ \quad + k_{2} L_{2} (L_{2} \theta_{x} - y_{1} ) - F_{y} (L_{2} - L_{5} ) = F_{impy} (L_{2} - L_{5} ), \hfill \\ \end{gathered}$$

12d $$\begin{gathered} J_{d1} \ddot{\theta }_{y} - J_{p1} \omega_{1} \dot{\theta }_{x} + c_{1} L_{1} (L_{1} \dot{\theta }_{y} - \dot{x}_{1} ) + c_{2} L_{2} (L_{2} \dot{\theta }_{y} + \dot{x}_{1} ) + k_{1} L_{1} (L_{1} \theta_{y} - x_{1} ) \hfill \\ \quad + k_{2} L_{2} (L_{2} \theta_{x} + x_{1} ) + F_{x} (L_{2} - L_{5} ) = - F_{impx} (L_{2} - L_{5} ), \hfill \\ \end{gathered}$$

12e $$m_{2} \ddot{x}_{2} + c_{3} (\dot{x}_{2} - L_{3} \dot{\phi }_{y} ) + k_{3} (x_{2} - L_{3} \phi_{y} ) - F_{x} = m_{2} e_{2} \omega_{2}^{2} \cos (\omega_{2} t) - m_{2} g,$$

12f $$m_{2} \ddot{y}_{2} + c_{3} (\dot{y}_{2} + L_{3} \dot{\phi }_{x} ) + k_{3} (y_{2} + L_{3} \phi_{x} ) - F_{y} = m_{2} e_{2} \omega_{2}^{2} \sin (\omega_{2} t),$$

12g $$J_{d2} \ddot{\phi }_{x} + J_{p2} \omega_{2} \dot{\phi }_{y} + c_{3} L_{3} (L_{3} \dot{\phi }_{x} + \dot{y}_{2} ) + k_{3} L_{3} (L_{3} \phi_{x} + y_{2} ) + F_{y} L_{4} = 0$$

12h $$J_{d2} \ddot{\phi }_{y} - J_{p2} \omega_{2} \dot{\phi }_{x} + c_{3} L_{3} (L_{3} \dot{\phi }_{y} - \dot{x}_{2} ) + k_{3} L_{3} (L_{3} \phi_{y} - x_{2} ) - F_{x} L_{4} = 0.$$

Make the above Equation dimensionless by introducing the following dimensionless parameters $\tau = \omega_{{1}} t$, $E_{1} = \frac{{e_{1} }}{{\delta_{0} }}$, $E_{2} = \frac{{e_{2} }}{{\delta_{0} }}$, $X_{1} = \frac{{x_{1} }}{{\delta_{0} }}$, $Y_{1} = \frac{{y_{1} }}{{\delta_{0} }}$, $X_{2} = \frac{{x_{2} }}{{\delta_{0} }}$, $Y_{2} = \frac{{y_{2} }}{{\delta_{0} }}$, $\Theta_{x} = \frac{l}{{\delta_{0} }}\theta_{x}$, $\Theta_{y} = \frac{l}{{\delta_{0} }}\theta_{y}$, $\Psi_{x} = \frac{l}{{\delta_{0} }}\psi_{x}$, $\Psi_{y} = \frac{l}{{\delta_{0} }}\psi_{y}$, $\lambda = \frac{{\omega_{2} }}{{\omega_{1} }}$, $\eta_{1} = \frac{{J_{{{\text{p}}_{1} }} }}{{J_{{{\text{d}}_{1} }} }}$, $\eta_{2} = \frac{{J_{{{\text{p}}_{2} }} }}{{J_{{{\text{d}}_{2} }} }}$, $L_{1} = \frac{{l_{1} }}{l}$, $L_{2} = \frac{{l_{2} }}{l}$, $L_{3} = \frac{{l_{3} }}{l}$, $L_{4} = \frac{{l_{4} }}{l}$, $L_{5} = \frac{{l_{5} }}{l}$, $C_{1} = \frac{{c_{1} }}{{m_{1} \omega_{1} }}$, $C_{2} = \frac{{c_{2} }}{{m_{1} \omega_{1} }}$, $C_{3} = \frac{{c_{1} l_{1} l}}{{J_{{{\text{d}}_{1} }} \omega_{1} }}$, $C_{4} = \frac{{c_{2} l_{2} l}}{{J_{{{\text{d}}_{1} }} \omega_{1} }}$, $C_{5} = \frac{{c_{3} }}{{m_{2} \omega_{1} }}$ $r_{1} = \sqrt {\frac{{\sum\limits_{i = 1}^{{\overline{N}}} {\left\{ {\left[ {X_{1} \left( i \right) - \overline{X}_{1} } \right]^{2} + \left[ {Y_{1} \left( i \right) - \overline{Y}_{1} } \right]^{2} } \right\}} }}{{\overline{N}}}}$, $r_{2} = \sqrt {\frac{{\sum\limits_{i = 1}^{{\overline{N}}} {\left\{ {\left[ {X_{2} \left( i \right) - \overline{X}_{2} } \right]^{2} + \left[ {Y_{2} \left( i \right) - \overline{Y}_{2} } \right]^{2} } \right\}} }}{{\overline{N}}}}$, $\overline{X}_{1}$, $\overline{Y}_{1}$, $\overline{X}_{2}$, $\overline{Y}_{2}$, $\overline{N}$, then Eq. (12) can be written as follows

13a $$\begin{gathered} X_{1}^{\prime \prime } + C_{1} \left( {X_{1}^{\prime } - L_{1} \Theta_{y}^{\prime } } \right) + C_{2} \left( {X_{1}^{\prime } + L_{2} \Theta_{y}^{\prime } } \right) + K_{1} \left( {X_{1} - L_{1} \Theta_{y} } \right) \hfill \\ \quad + K_{2} \left( {X_{1} + L_{2} \Theta_{y} } \right) = E_{1} \cos \tau - \frac{{\hat{F}_{X} }}{{m_{1} \omega_{1}^{2} }} - \frac{g}{{\omega_{1}^{2} \delta_{0} }} - \frac{{f_{bx} }}{{m_{1} \omega_{1}^{2} }} - \frac{{f_{impx} }}{{m_{1} \omega_{1}^{2} }}, \hfill \\ \end{gathered}$$

13b $$\begin{gathered} Y^{\prime\prime}_{1} + C_{1} \left( {Y^{\prime}_{1} + L_{1} \Theta^{\prime}_{x} } \right) + C_{2} \left( {Y^{\prime}_{1} - L_{2} \Theta^{\prime}_{x} } \right) + K_{1} \left( {Y_{1} + L_{1} \Theta_{x} } \right) \hfill \\ \quad + K_{2} \left( {Y_{1} - L_{2} \Theta_{x} } \right) = E_{1} \sin \tau - \frac{{\hat{F}_{Y} }}{{m_{1} \omega_{1}^{2} }} - \frac{{f_{bx} }}{{m_{1} \omega_{1}^{2} }} + \frac{{f_{impy} }}{{m_{1} \omega_{1}^{2} }}, \hfill \\ \end{gathered}$$

13c $$\begin{gathered} \Theta^{\prime\prime}_{x} + \eta_{1} \Theta^{\prime}_{y} + C_{3} \left( {Y^{\prime}_{1} + L_{1} \Theta^{\prime}_{x} } \right) - C_{4} \left( {Y^{\prime}_{1} - L_{2} \Theta^{\prime}_{x} } \right) + K_{3} \left( {Y_{1} + L_{1} \Theta_{x} } \right) \hfill \\ \quad - K_{4} \left( {Y_{1} - L_{2} \Theta_{x} } \right) = \frac{{\hat{F}_{Y} l\left( {l_{2} - l_{5} } \right)}}{{J_{{d_{1} }} \omega_{1}^{2} }} - \frac{{f_{impy} \left( {l_{2} - l_{5} } \right)}}{{J_{{d_{1} }} \omega_{1}^{2} }}, \hfill \\ \end{gathered}$$

13d $$\begin{gathered} \Theta^{\prime\prime}_{y} - \eta_{1} \Theta^{\prime}_{x} - C_{3} \left( {X^{\prime}_{1} - L_{1} \Theta^{\prime}_{y} } \right) + C_{4} \left( {X^{\prime}_{1} + L_{2} \Theta^{\prime}_{y} } \right) - K_{3} \left( {X_{1} - L_{1} \Theta_{y} } \right) \hfill \\ \quad + K_{4} \left( {X_{1} + L_{2} \Theta_{y} } \right) = - \frac{{\hat{F}_{X} l\left( {l_{2} - l_{5} } \right)}}{{J_{{d_{1} }} \omega_{1}^{2} }} - \frac{{f_{impx} \left( {l_{2} - l_{5} } \right)}}{{J_{{d_{1} }} \omega_{1}^{2} }}, \hfill \\ \end{gathered}$$

13e $$X^{\prime\prime}_{2} + C_{5} \left( {X^{\prime}_{2} - L_{3} \Psi^{\prime}_{y} } \right) + K_{5} \left( {X_{2} - L_{3} \Psi_{y} } \right) = \lambda^{2} E_{2} \cos \left( {\lambda \tau } \right) + \frac{{\hat{F}_{X} }}{{m_{2} \omega_{1}^{2} }} - \frac{g}{{\omega_{1}^{2} \delta_{0} }},$$

13f $$Y^{\prime\prime}_{2} + C_{5} \left( {Y^{\prime}_{2} + L_{3} \Psi^{\prime}_{x} } \right) + K_{5} \left( {Y_{2} + L_{3} \Psi_{x} } \right) = \lambda^{2} E_{2} \sin \left( {\lambda \tau } \right) + \frac{{\hat{F}_{Y} }}{{m_{2} \omega_{1}^{2} }},$$

13g $$\Psi^{\prime\prime}_{x} + \lambda \eta_{2} \Psi^{\prime}_{y} + C_{6} \left( {Y^{\prime}_{2} + L_{3} \Psi^{\prime}_{x} } \right) + K_{6} \left( {Y_{2} + L_{3} \Psi_{x} } \right) = - \frac{{\hat{F}_{Y} l_{4} l}}{{J_{{d_{2} }} \omega_{1}^{2} }},$$

13h $$\Psi^{\prime\prime}_{y} - \lambda \eta_{2} \Psi^{\prime}_{x} - C_{6} \left( {X^{\prime}_{2} - L_{3} \Psi^{\prime}_{y} } \right) - K_{6} \left( {X_{2} - L_{3} \Psi_{y} } \right) = \frac{{\hat{F}_{X} l_{4} l}}{{J_{{d_{2} }} \omega_{1}^{2} }}$$

where the dimensionless restoring force is as follows

14 $$\left( \begin{gathered} \hat{F}_{X} \hfill \\ \hat{F}_{Y} \hfill \\ \end{gathered} \right) = \left( \begin{gathered} \frac{{F_{x} }}{{\delta_{0} }} \hfill \\ \frac{{F_{y} }}{{\delta_{0} }} \hfill \\ \end{gathered} \right) = K_{b} \delta_{0}^{1/9} \sum\limits_{k = 1}^{{N_{b} }} {\hat{\delta }_{k}^{10/9} } G(\hat{\delta }_{k} )\left( \begin{gathered} \cos \hat{\theta }_{k} \hfill \\ \sin \hat{\theta }_{k} \hfill \\ \end{gathered} \right).$$

The dimensionless impact force of inter-shaft bearing is as follows

15 $$\left( \begin{gathered} f_{impx} \hfill \\ f_{impy} \hfill \\ \end{gathered} \right) = \left( {\frac{{F_{impx} }}{{\delta_{0} }},\frac{{F_{impy} }}{{\delta_{0} }}} \right)^{T} { = }\frac{{F_{imp} }}{{\delta_{0} }}\left( \begin{gathered} \cos \theta_{e} \hfill \\ \sin \theta_{e} \hfill \\ \end{gathered} \right).$$

The aerodynamic excitation force is as follows

16 $$\left( \begin{gathered} f_{bx} \hfill \\ f_{by} \hfill \\ \end{gathered} \right) = \left( {\frac{{F_{bx} }}{{\delta_{0} }},\frac{{F_{by} }}{{\delta_{0} }}} \right)^{T} = \frac{{F_{b} }}{{\delta_{0} }}\left( \begin{gathered} \cos \hat{\theta }_{k} \hfill \\ \sin \hat{\theta }_{k} \hfill \\ \end{gathered} \right).$$

The dimensionless position of the *k*th roller is $\hat{\theta }_{k} = 2\pi (k - 1)/N_{b} + \hat{\omega }_{c} t$, and the dimensionless angular velocity of the cage is $\hat{\omega }_{c} = \frac{{\omega_{c} }}{{\omega_{1} }}$, the dimensionless contact deformation is

17 $$\begin{gathered} \hat{\delta }_{k} = \frac{{\delta_{k} }}{{\delta_{0} }} = \{ [X_{1} + \Theta_{y} (L_{2} - L_{5} )] - (X_{2} + \Psi_{y} L_{4} )\} \cos \theta_{k} \hfill \\ + \{ [Y_{1} - \Theta_{x} (L_{2} - L_{5} )] - (Y_{2} - \Psi_{x} L_{4} )\} \sin \theta_{k} - 1\quad (k = 1,2, \ldots ,N_{b} ). \hfill \\ \end{gathered}$$

Since the dual-rotor system is subjected to dual-frequency unbalanced excitation from the HP and LP rotors, there are at least two frequency components. The amplitude in the amplitude-frequency curve is represented by the effective value^17^. The LP and HP rotors' amplitudes are as follows

18a $$r_{1} = \sqrt {\frac{{\sum\limits_{i = 1}^{{\overline{N}}} {\left\{ {\left[ {X_{1} \left( i \right) - \overline{X}_{1} } \right]^{2} + \left[ {Y_{1} \left( i \right) - \overline{Y}_{1} } \right]^{2} } \right\}} }}{{\overline{N}}}} ,$$

18b $$r_{2} = \sqrt {\frac{{\sum\limits_{i = 1}^{{\overline{N}}} {\left\{ {\left[ {X_{2} \left( i \right) - \overline{X}_{2} } \right]^{2} + \left[ {Y_{2} \left( i \right) - \overline{Y}_{2} } \right]^{2} } \right\}} }}{{\overline{N}}}} ,$$

where $\overline{X}_{1}$, $\overline{Y}_{1}$, $\overline{X}_{2}$, $\overline{Y}_{2}$ respectively represent the average values of the corresponding dimensionless responses in horizontal and vertical directions of LP and HP rotors, $\overline{N}$ denoted discretization points' number.

### Synchronous impact

**Figure 6:**
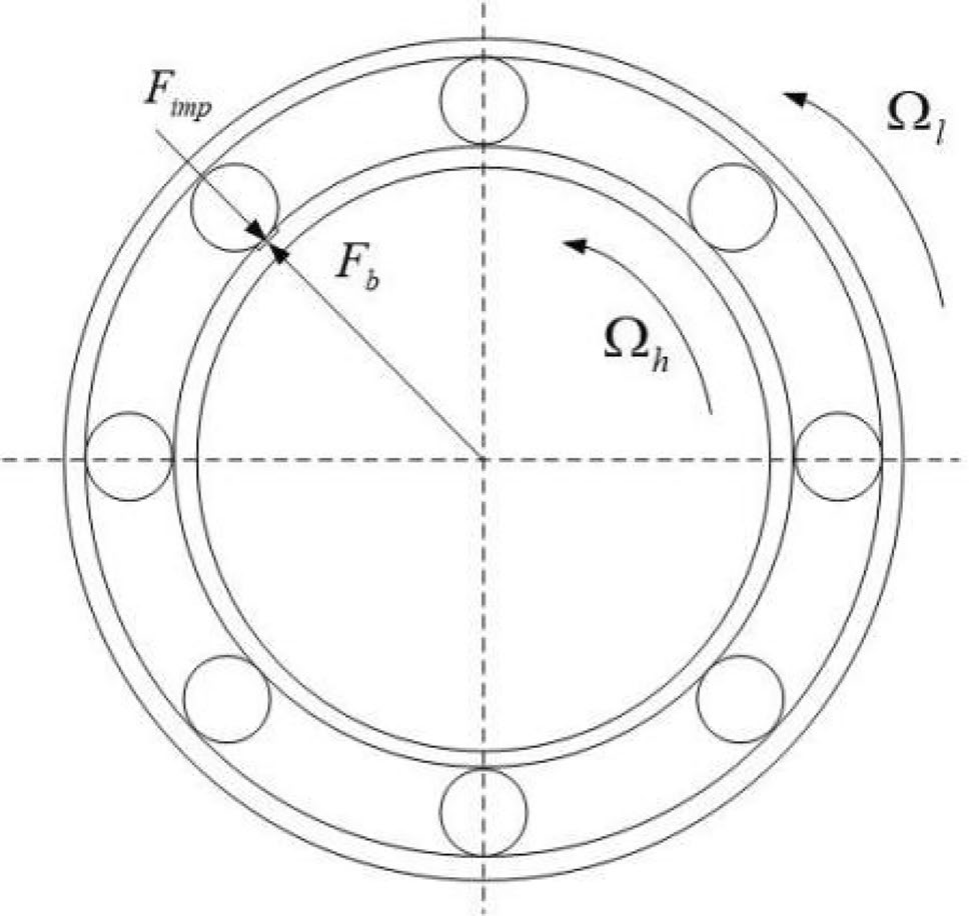
Model of inter-shaft bearing's load.

During engine operation, when a defect of the bearing contacts the rollers of the bearing, the fan blades will also be impacted by the rotating aerodynamic excitation force due to dynamic and static interference. They will be transmitted to the inter-shaft bearing. At this time, the dynamic load at the bearing defect is the superposition of the impact force of the rollers and the aerodynamic excitation force^38^. Figure 6 is a schematic diagram of the impact force generated by the contact between the rollers and a particular position of the inner race.

The bearing’s typical characteristic frequency formulas indicate that the inner and outer races' contact frequencies of the inter-shaft bearing are

19a $$f_{inner} = \frac{1}{2 \times 2\pi }\left| {\Omega_{h} - \Omega_{l} } \right|\left( {1 + \frac{d}{{D_{m} }}\cos \alpha } \right)N_{b} ,$$

19b $$f_{outer} = \frac{1}{2 \times 2\pi }\left| {\Omega_{h} - \Omega_{l} } \right|\left( {1 - \frac{d}{{D_{m} }}\cos \alpha } \right)N_{b} ,$$

where *N*_*b*_ is the number of rollers, *d* is the diameter of the roller, *D*_*m*_ is the bearing pitch diameter, and *α* is the contact angle.

According to the formula (10), the main frequency of aerodynamic excitation force is an integer multiple of the number of fan blades *Z*_*b*_ and the rotational speed of the bearing’s inner race, the load of the raceway consists of two parts: the impact force of the rollers and the aerodynamic excitation force. When the frequency of the aerodynamic excitation force is not close to the characteristic frequency of the inner race, that means

20 $$\left\{ \begin{gathered} f_{inner} \ne \frac{{KZ_{b} \Omega_{l} }}{2\pi } \hfill \\ f_{outer} \ne \frac{{KZ_{b} \Omega_{l} }}{2\pi } \hfill \\ \end{gathered} \right.\quad \left( {{\text{K}} = { 1},{2},{3} \ldots } \right).$$

The impact force at the defect will not always be equal to the sum of the impact force of the roller and the amplitude of the aerodynamic excitation force; however, when the aerodynamic excitation force and the impact force of the roller are in the same frequency and phase, that is

21 $$\left\{ \begin{gathered} f_{inner} \approx \frac{{KZ_{b} \Omega_{l} }}{2\pi } \hfill \\ f_{outer} \approx \frac{{KZ_{b} \Omega_{l} }}{2\pi } \hfill \\ \end{gathered} \right.\quad \left( {{\text{K}} = { 1},{2},{3} \ldots } \right).$$

The impact force at the defect is the sum of the impact force of the rollers and the aerodynamic excitation force, which continuously acts on the same point. This phenomenon is called synchronous impact. Under the action of synchronous impact, the dynamic load on a particular point of the inter-shaft bearing increases significantly, which is easy to cause damage to the raceway and reduces the life of the inter-shaft bearing.

## Numerical results and discussions

### Calculation of synchronous impact conditions

The LP and HP rotors' amplitude-frequency vibration curves are drawn according to the numerical results obtained by the fourth-order Runge–Kutta method. The system parameters are as follows^32^, Table 1 shows the parameters of the dual-rotor system, Table 2 shows the parameters of the three support bearings, Table 3 shows the parameters of the inter-shaft bearing.

**Table 1:** Parameters of the dual-rotor system.

Parameter of LP rotor	Value	Parameter of HP rotor	Value
Mass *m*_1_ (kg)	97.37	Mass *m*_2_ (kg)	108.30
Polar moment *J*_*p*1_ (kg m^2^)	3.69	Polar moment *J*_*p*2_ (kg m^2^)	4.01
Diameter moment *J*_*d*1_ (kg m^2^)	1.85	Diameter moment *J*_*d*2_ (kg m^2^)	2
Eccentricity *e*_1_ (µm)	1	Eccentricity *e*_2_ (µm)	0.5

**Table 2:** Parameters of the three support bearings.

Support bearing number	Stiffness (N/m)	Damping (Ns/m)
No. 1	6 × 10^7^	655
No. 2	6 × 10^7^	655
No. 3	6 × 10^7^	655

**Table 3:** Parameters of the inter-shaft bearing.

Parameter	Value
Radiuses of inner race *r*_*i*_ (mm)	58
Radiuses of outer race *r*_*o*_ (mm)	6 × 10^7^
Number of balls *N*_*b*_	6 × 10^7^
Contact stiffness *K*_*b*_ (N/m^10/9^)	10^8^
Bearing clearance *δ*_*0*_ (μm)	1
Depth of defect *δ*_*e*_ (μm)	20
Defect span *l*_*e*_ (mm)	10

System structure parameters are as follows.

*l*_1_ = 0. 92 m, *l*_2_ = 1. 11 m, *l*_3_ = 0. 51 m, *l*_4_ = 0. 62 m, *l*_5_ = 0. 0995 m, *Z*_*b*_ = 8, *F*_0_ = 100 N.

According to the determination formula of the system's synchronous impact (19), both ends of the determination formula are divided by *Ω*_*l*_ at the same time to obtain

22a $$\frac{1}{2 \times 2\pi }\left| {\lambda - 1} \right|\left( {1 + \frac{d}{Dm}\cos \alpha } \right)z \approx \frac{{KZ_{b} }}{2\pi }$$

22b $$\frac{1}{2 \times 2\pi }\left| {\lambda - 1} \right|\left( {1 - \frac{d}{Dm}\cos \alpha } \right)z \approx \frac{{KZ_{b} }}{2\pi }$$

Substituting the system’s parameters into Eqs. (22a) and (22b) to make the two sides of the determination equation equal, the relationship between K and the speed ratio is obtained in Table 4.

**Table 4:** Speed ratio of HP and LP under synchronous impact.

	K	1	2	3
Rotate in the same direction	Speed ratio (inner race)	1.75	2.50	3.25
	Speed ratio (outer race)	1.85	2.71	3.57

As shown in the table above, when the speed ratio of the HP and LP rotor meets certain conditions, the speed ratio of the system meets the conditions of synchronous impact in the operation process, synchronous impact phenomenon will occur at this time, resulting in the system load concentration and sudden increase of force, making the system vulnerable to irreversible damage at this time.

### Vibration response of the dual-rotor system under synchronous impact

Consider the dual-rotor inter-shaft bearing system with a defect in the inner race whose HP and LP rotors rotate in the same direction as an example. When the system’s speed ratio gradually approaches the determination condition of synchronous impact, when $\lambda$ close to 1.75, synchronous impact occurs.

**Figure 7:**
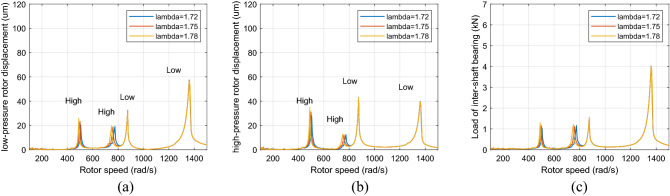
Amplitude-frequency response curve without defect (λ = 1. 72, 1. 75, 1. 78, F_0_ = 0, A_max_ = 0) (**a**) LP rotor (**b**) HP rotor (**c**) inter-shaft bearing’s load.

**Figure 8:**
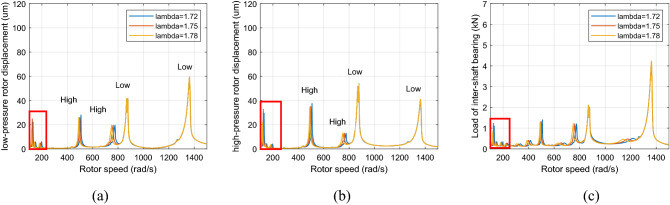
Amplitude-frequency response curve with defect (λ = 1. 72, 1. 75, 1. 78, F_0_ = 0, A_max_ = 200) (**a**) LP rotor (**b**) HP rotor (**c**) inter-shaft bearing’s load.

**Figure 9:**
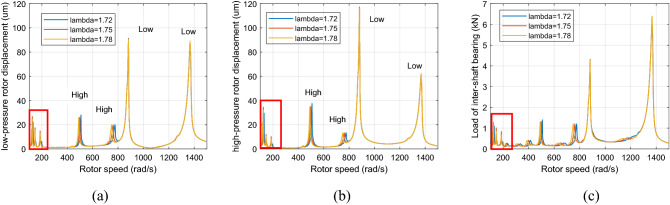
Amplitude-frequency response curve with defect (λ = 1. 72, 1. 75, 1. 78, F_0_ = 100, A _max_ = 200) (**a**) LP rotor (**b**) HP rotor (**c**) inter-shaft bearing’s load.

Figure 7 shows the healthy dual-rotor system’s amplitude-frequency curve when the aerodynamic excitation force and roller impact force are set to 0. Figures 8 and 9 show the dual-rotor system's amplitude-frequency curve considering the defect in the inter-shaft bearing's inner race when the aerodynamic excitation force is set to 0 and 100 N in the low-frequency region, respectively. The abscissa in the figure is the speed of the LP rotor (rad/s), and the ordinate is the amplitude of the rotor at the corresponding rate (μm). Figures 8 and 9 show that the aerodynamic excitation force *F*_0_ rises from 0 to 100 N, and the primary resonance peak of the HP rotor does not change significantly when the speed ratio is 1.72, 1.75, and 1.78. The primary resonance peak of the LP rotor increases considerably with the increase of aerodynamic excitation force because the aerodynamic excitation force mainly affects the LP rotor in the model of the dual-rotor system. The primary resonance peak of HP appears at 500 rad/s, the primary resonance peak of LP appears at 882 rad/s, and the location of the primary resonance peak of the HP and LP rotor *Ω*_*h*_/*Ω*_*l*_ = 1. 76, which is close to the HP and LP rotor speed ratio. Take λ = 1. 75 as an example, the amplitude of the first-order primary resonance peak of the LP rotor increases from 40.7 to 91 μm, and the amplitude of the second-order primary resonance peak of the LP rotor increases from 59.2 to 89.1 μm. The situation of the HP rotor is similar.

**Figure 10:**
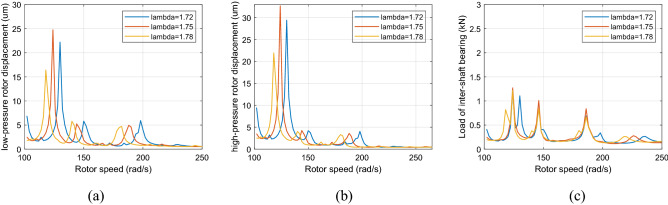
Partial enlarged view of Fig. 8 (λ = 1. 72, 1. 75, 1. 78, F_0_ = 0, A _max_ = 200) (**a**) LP rotor (**b**) HP rotor (**c**) inter-shaft bearing’s load.

**Figure 11:**
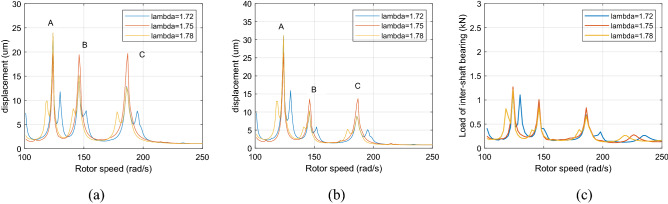
Partial enlarged view of Fig. 9 (λ = 1. 72, 1. 75, 1. 78, F_0_ = 100, A _max_ = 200) (**a**) LP rotor (**b**) HP rotor (**c**) inter-shaft bearing’s load.

Figure 10 and 11 show that when the rotor speed *Ω*_*l*_ is set to 124, 146, and 186 rad/s under the aerodynamic excitation force, three resonance peaks of system A, B and C increase, which can be called aerodynamic resonance. When the value of λ is 1.75, relative to the value of 1.72 and 1.78, the amplitude of B and C aerodynamic resonance is the highest, and the aerodynamic resonance peak of A is lowest, amplitudes of A, B and C are 21.3, 19.2, 17.3 μm respectively. Figures 10c and 11c show that aerodynamic excitation force amplifies the load of the inter-shaft bearing. While the system runs under the synchronous impact (λ = 1.75), the inter-shaft bearing's load is most significant at the aerodynamic resonances B and C compared to the aerodynamic resonances A. The bearing’s load reach1.27, 1.01 and 0.84 KN respectively, which is easy to cause the phenomenon of load concentration and increases the probability of bearing damage. Both the bearing defect and the roller impact force are considered in the system. The amplitude of the aerodynamic resonance increases significantly when the synchronous impact phenomenon occurs. The speed of the high-pressure rotor rises with the increase of the HP/LP speed ratio when the low-pressure rotor speed is unchanged, which leads to the increase of the fault frequency of the inter-shaft bearing. Figure 10 in this paper shows that the resonance generated by the roller impact force shifts to the left with the increase of the HP/LP speed ratio, which means the resonance occurs earlier. When synchronous impact occurs, the resonance generated by the roller impact force and the aerodynamic resonance are coupled at the same rotation speed, increasing the combined resonance generated during the synchronous impact. Therefore, the bearing defects are considered because of the amplitude amplification caused by the synchronous impact. Compared with a healthy dual-rotor system without faults, the synchronous impact phenomenon of the system is more pronounced. As the aerodynamic excitation force obviously increase the amplitude of the primary resonance caused by the LP rotor of the dual-rotor system, and excite three aerodynamic resonances in the low-frequency region, the instability of the system is amplified.

**Figure 12:**
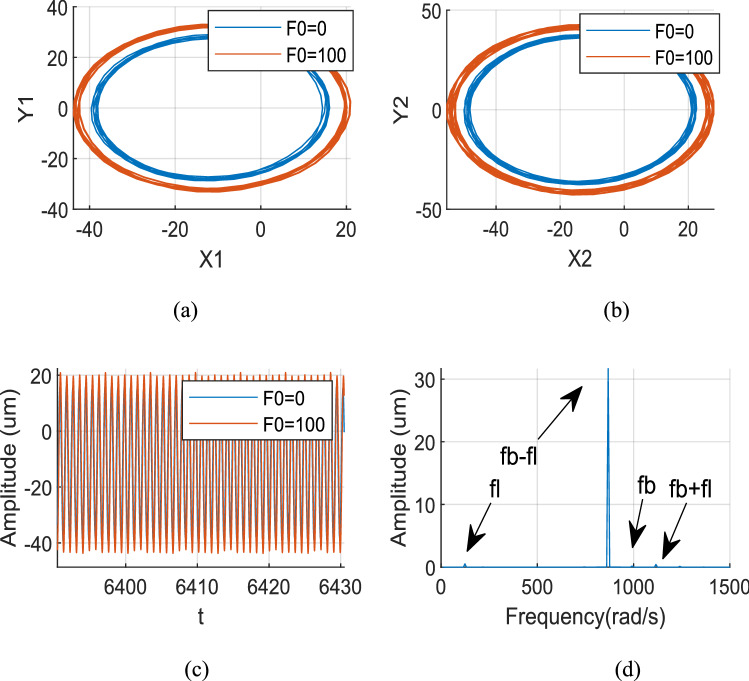
Aerodynamic resonance A of LP rotor (λ = 1. 75, ω_l_ = 124 rad/s) (**a**) Orbit diagram of LP rotor (**b**) Orbit diagram of HP rotor (**c**) Time history (**d**) Frequency spectrum.

**Figure 13:**
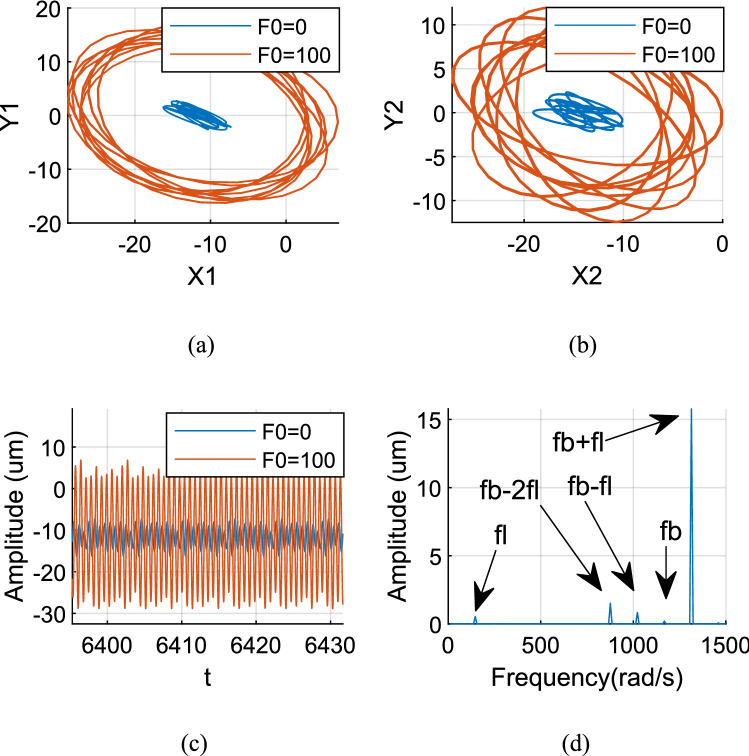
Aerodynamic resonance B of LP rotor (λ = 1. 75, ω_l_ = 146 rad/s) (a) Orbit diagram of LP rotor (b) Orbit diagram of HP rotor (c) Time history (d) Frequency spectrum.

**Figure 14:**
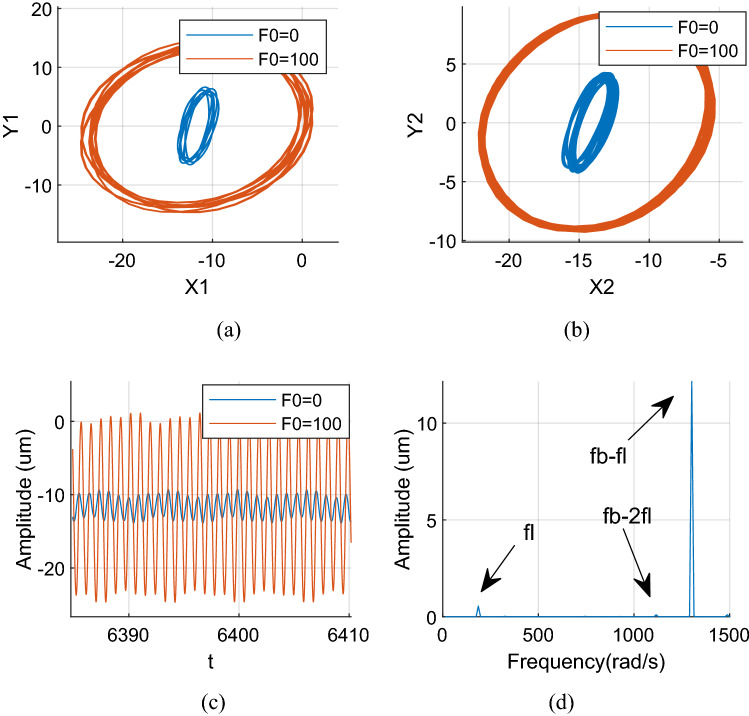
Aerodynamic resonance C of LP rotor (λ = 1. 75, ω_l_ = 186 rad/s) (**a**) Orbit diagram of LP rotor (**b**) Orbit diagram of HP rotor (**c**) Time history (**d**) Frequency spectrum.

Figures 12, 13 and 14 show the axis trajectory and time history images when *Ω*_*l*_ is taken at 124, 146, 186, and 882 rad/s, λ = 1.75, and *F*_0_ is taken at 0 and 100 N, respectively. It shows that the range movement of the axis trajectory of the system is enlarged under the action of aerodynamic excitation force.

Figure 12 shows that when *Ω*_*l*_ is set to 124 rad/s, the orbit of the centre O_2_ of peak A is an ellipse, and the response is a periodic signal. The resonance of the system is excited by multiple excitation frequencies, which include the frequency *f*_*l*_ excited by the LP rotor, the frequencies of aerodynamic excitation force *f*_*b*_ and roller impact force *f*_*imp*_ (*f*_*b*_ = *f*_*imp*_ when λ = 1.75), while their combined frequencies *f*_*b *_− *f*_*l*_, *f*_*b*_ + *f*_*l*_ are included in the spectrum diagram, among these frequencies *f*_*b *_− *f*_*l*_ plays a most critical role.

Figure 13 shows that when *Ω*_*l*_ is set to 146 rad/s, the orbit of the centre O_2_ of peak A is an ellipse, and the response is a quasi-periodic signal. The resonance of the system is excited by multiple excitation frequencies, which include the frequency *f*_*l*_ excited by the LP rotor, the frequencies of aerodynamic excitation force *f*_*b*_ and roller impact force *f*_*imp*_ (*f*_*b*_ = *f*_*imp*_ when λ = 1.75), while their combined frequencies *f*_*b *_− 2*fl*, *f*_*b *_− *f*_*l*_, *f*_*b*_ + *f*_*l*_ are included in the spectrum diagram, among these frequencies *f*_*b*_ + *f*_*l*_ plays a most important role.

Figure 14 shows that when *Ω*_*l*_ is set to 186 rad/s, the orbit of the centre O_2_ of peak A is an ellipse, and the response is a quasi-periodic signal. The resonance of the system is excited by multiple excitation frequencies, which include the frequency *f*_*l*_ excited by the LP rotor, the frequencies of aerodynamic excitation force *f*_*b*_ and roller impact force *f*_*imp*_ (*f*_*b*_ = *f*_*imp*_ when λ = 1.75), meanwhile, their combined frequencies *f*_*b *_− 2*fl*, *f*_*b *_− *f*_*l*_ are included in the spectrum diagram, among these frequencies *f*_*b *_− *f*_*l*_ plays a most important role.

**Figure 15:**
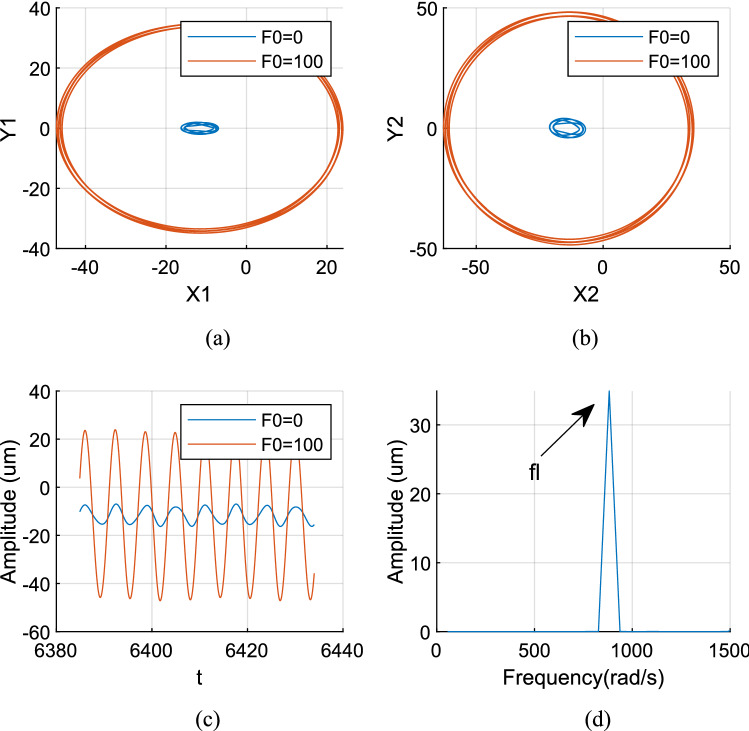
First order primary resonance of LP rotor (λ = 1. 75, ω_l_ = 882 rad/s) (**a**) Orbit diagram of LP rotor (**b**) Orbit diagram of HP rotor (**c**) Time history (**d**) Frequency spectrum.

Figure 15 shows that when *Ω*_*l*_ is set to 882 rad/s, the LP rotor’s rotation frequency coincides with the system's natural frequency, which strictly stimulates the system’s first-order primary resonance. Therefore, the LP rotor's rotation frequency *f*_*l*_ in the spectrum diagram is the primary reason which causes a resonance of the system. At this time, the orbit track of the LP rotor becomes a regular ellipse; its response becomes a harmonic signal.

### Influence of initial phase difference of local defect

**Figure 16:**
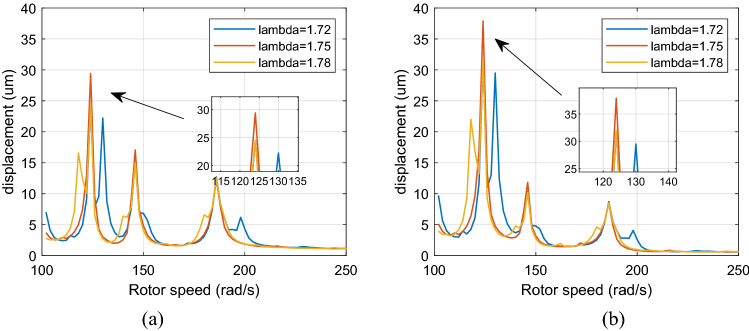
Amplitude-frequency response curve (λ = 1. 72, 1. 75, 1. 78, F_0_ = 100, A_max_ = 200, ψ = π) (**a**) LP rotor (**b**) HP rotor.

The initial phase difference of the dual-rotor system is set π (the direction of the aerodynamic excitation force is the same as the direction of the roller impact force), similar to the case that the phase difference is set 0, the aerodynamic resonances are shown in the amplitude-frequency response under aerodynamic excitation force. When the speed ratio λ is 1.75, the aerodynamic resonance A is the largest, while aerodynamic resonances B and C are minor (Fig. 16).

### Influence of rotor eccentricity on the system

**Figure 17:**
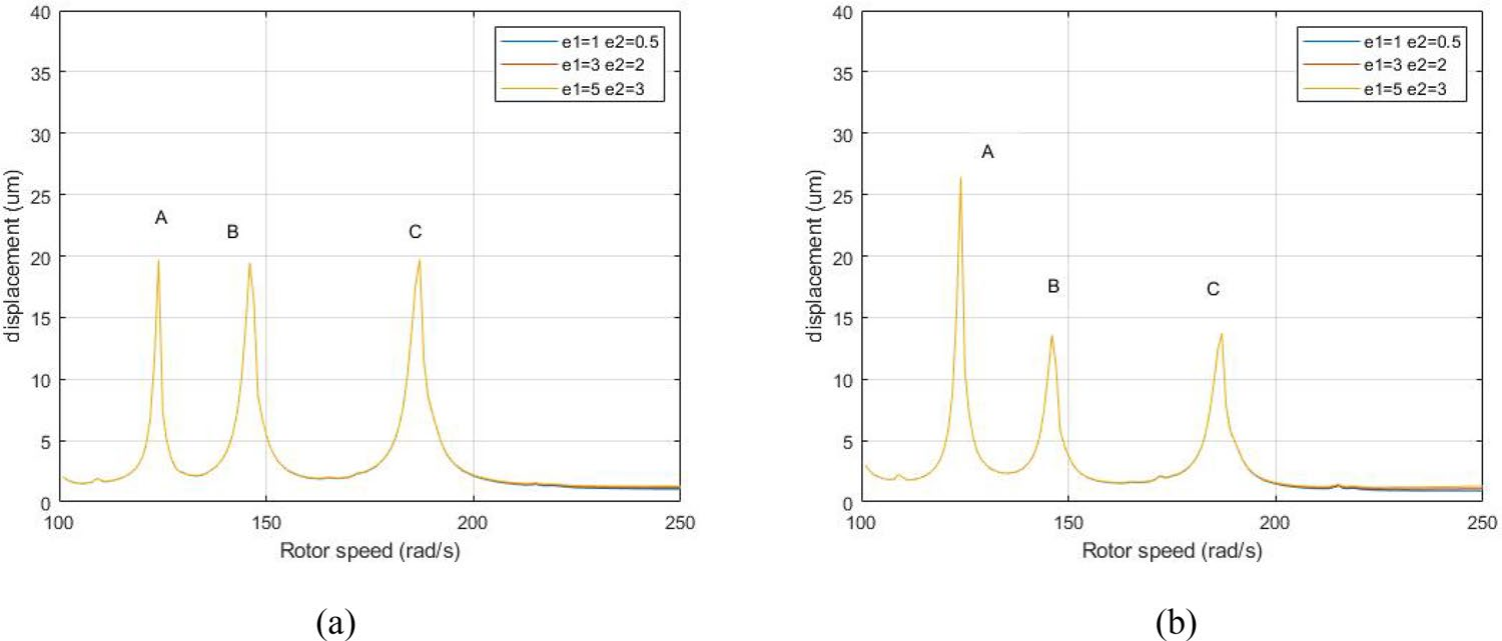
Amplitude-frequency response curve (λ = 1. 75, F_0_ = 100, A _max_ = 200) (**a**) LP rotor (**b**) HP rotor.

Figure 17 identifies the system’s amplitude-frequency response with different eccentricities when λ = 1. 75. The eccentricities of the LP and HP rotor are e_1_ = 1 μm, e_2_ = 0. 5 μm, e_1_ = 3 μm, e_2_ = 2 μm, e_1_ = 5 μm, e_2_ = 3 μm respectively.

As demonstrated by Fig. 17, when the system's eccentricity of the LP rotor and HP rotor increases, the peaks of A, B and C of the dual-rotor system do not transform. The resonance position of the system does not change, indicating that the resonance is not caused by unbalanced excitation.

### Influence of inter-shaft bearing clearance

**Figure 18:**
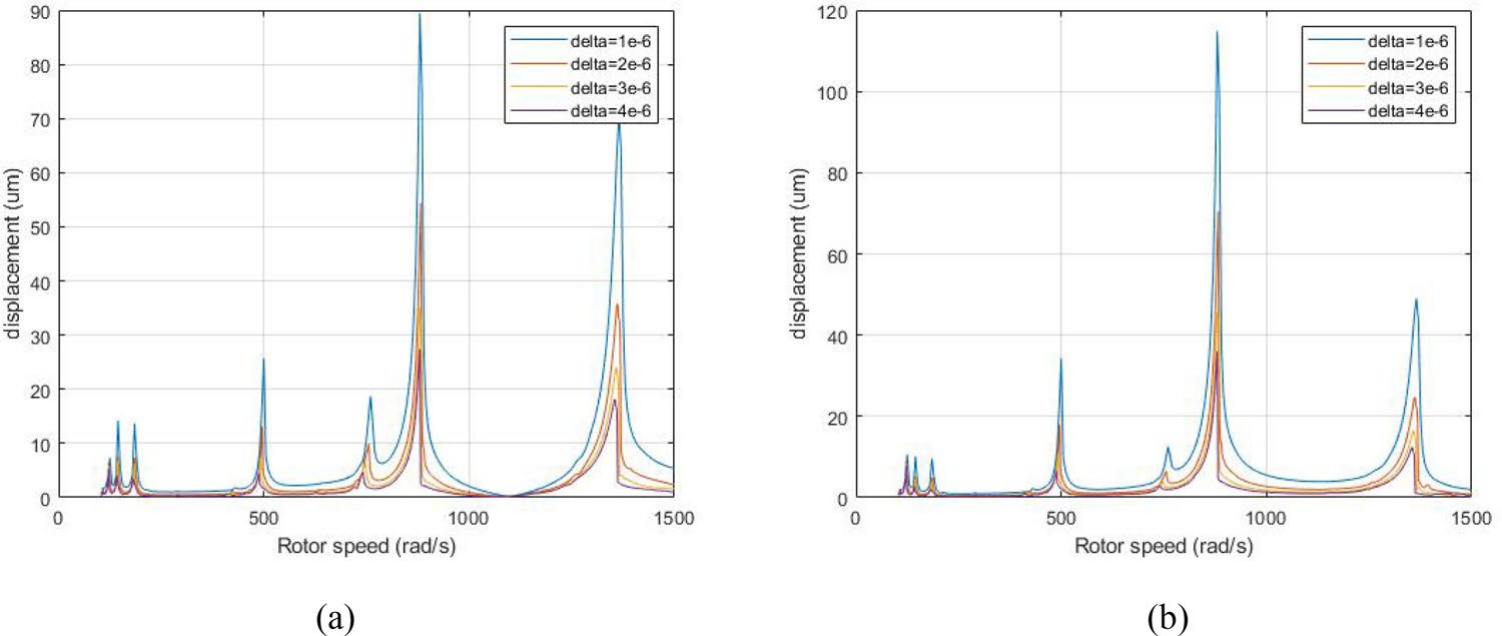
Amplitude-frequency response curve (λ = 1. 75, F_0_ = 100, A _max_ = 200) (**a**) LP rotor (**b**) HP rotor.

Figure 18 is a comparison diagram of amplitude-frequency curves when the system is subjected to aerodynamic excitation force at λ = 1. 75, and the clearance of the system are δ_0_ = 1, 2, 3, 4 μm.

Figure 18 demonstrates that the peaks of A, B and C and primary resonance of the system’s first-order and second-order will decrease significantly with the increase of clearance of the system's inter-shaft bearing, and the LP rotor's resonance position will not shift while the resonance frequencies of A, B, C and the HP rotor's resonance position is moved to the right when the inter-shaft bearing's clearance decrease.

### Influence of stiffness of supports

**Figure 19:**
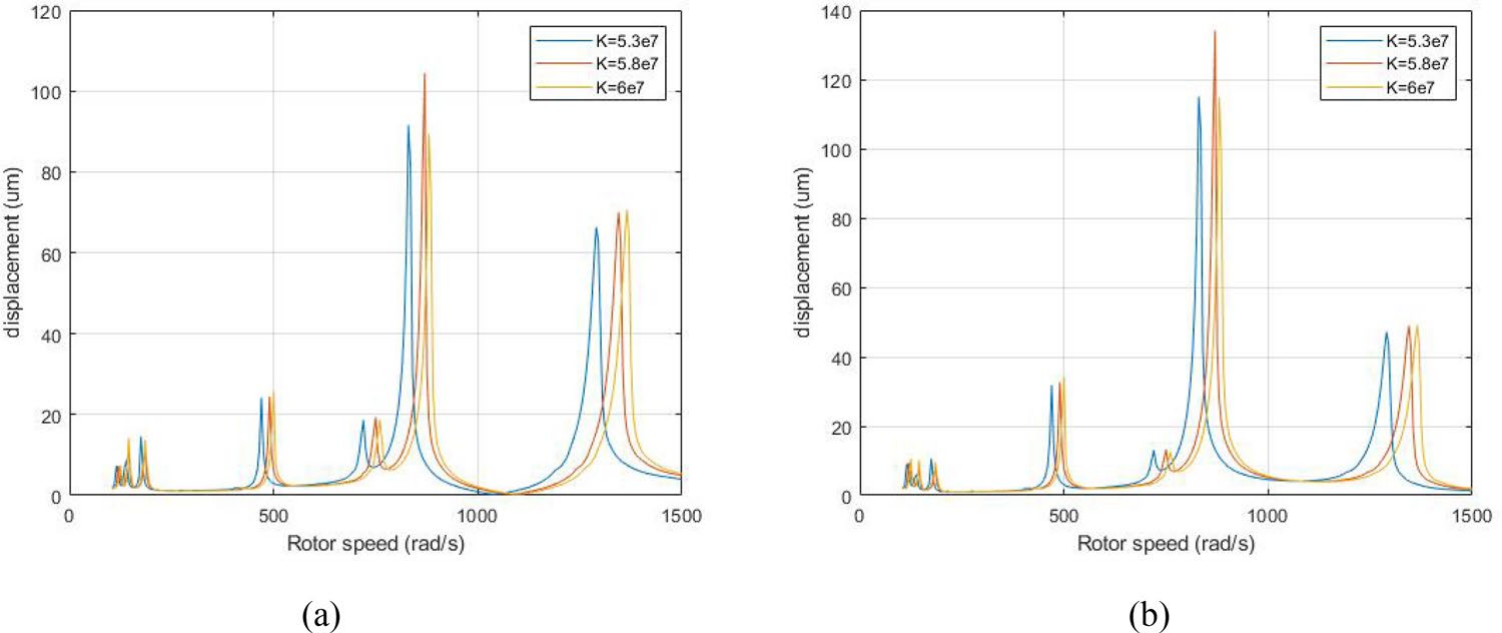
Amplitude-frequency response curve (λ = 1. 75, F_0_ = 100, A _max_ = 200) (**a**) LP rotor (**b**) HP rotor.

Figure 19 is a comparison diagram of the amplitude-frequency curve of the system support under different stiffness when the system is subjected to aerodynamic excitation force when λ = 1.75. The stiffness of the system's support bearings is K = 5.3 × 10^7^, 5.8 × 10^7^, 6 × 10^7^ N/m.

Figure 19 illustrates that as the rigidity of the system support increases, the amplitude of ABC peaks and primary resonance of the system's first-order and second-order slightly increase with the increase of the support rigidity; all the peaks of resonance positions of the system are shifted to the right together.

### Influence of damping of supports

**Figure 20:**
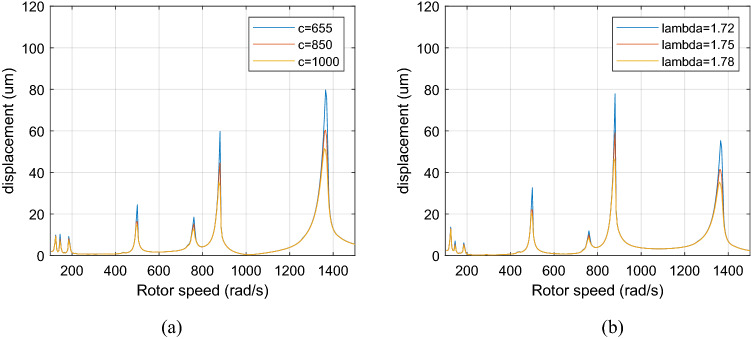
Amplitude-frequency response curve (λ = 1. 75, F_0_ = 100, A _max_ = 200) (**a**) LP rotor (**b**) HP rotor.

Figure 20 displays a comparison graph of the system's amplitude-frequency curves with different damping when the dual-rotor system is subjected to aerodynamic excitation force when λ = 1.75. The system’s damping is respectively taken as $c$ = 655, 850, and 1000 Ns/m.

Figure 20 illustrates that the amplitudes of all the resonance peaks of the system decrease considerably with the damping increase, and the resonance position of the system does not shift, which indicates that increasing the damping may reduce the resonance of the system.

## Conclusions

In this paper, the model of a dual-rotor system considering the impact force of the inter-shaft bearing and the aerodynamic excitation force has been formulated. It effectively supplements the dynamic characteristics of the dual-rotor system affected by aerodynamic forces. The amplitude-frequency responses of the two rotors and the dynamic load of the inter-shaft bearing have been obtained by numerical simulations. The nonlinear dynamic characteristics of the system affected by parameters such as phase difference of local defect, rotor eccentricity of system, clearance of inter-shaft bearing, and stiffness and damping of supports have been discussed in detail. The conclusions are as follows:

1. When the excitation frequency of the aerodynamic excitation force of the fan blade is close to the inter-shaft bearing's characteristic frequency, aerodynamic excitation force and roller impact force are superimposed, resulting in the occurrence of the synchronous impact phenomenon. It is demonstrated that the dual-rotor system's dynamic characteristic at the primary resonance caused by the LP rotor increases significantly; in contrast, the primary resonance caused by the HP rotor will not be changed. Consequently, the aerodynamic excitation force mainly affects the LP rotor.
2. Under the excitation of the aerodynamic force, the combination resonances of LP rotation frequency, aerodynamic excitation force frequency and roller impact force frequency may take place. Three resonance peaks emerge significantly in the low-frequency region of the dual-rotor system's amplitude-frequency response, increasing considerably under synchronous impact. It shows that the synchronous impact phenomenon can effectively improve the amplitude of the system at specific frequencies, leading to the increase of the load of the inter-shaft bearing and the instability of the system.
3. Under the condition of synchronous impact, the inter-shaft bearing's dynamic load increases significantly at the three emerged combination resonances. Additionally, the primary resonance caused by the LP rotor rises with the aerodynamic force increase, demonstrating that the LP rotor is mainly affected by aerodynamic exciting force.
4. The three emerged combination resonances are dominated by the aerodynamic excitation and roller impact forces. Still, they are not changed with the eccentricities of the HP and LP rotors of the dual-rotor system. According to the analysis results, the three additional amplitudes are combination resonances of the frequency of the aerodynamic excitation force, impact force and the rotation of the LP rotor.
5. The amplitude frequency response of the three emerged combination resonances is sensitive to the intersection angle between the directions of aerodynamic excitation force and roller impact force. When the intersection angle is π rad/s, one of the resonance peaks increases obviously, while the other two decrease slightly.
6. To reduce the three emerged combination resonances as well as the inter-shaft bearing's dynamic load, it is suggested to reduce the aerodynamic excitation force, reduce the roller impact force, reduce the stiffness of supports bearing, increase the damping of the dual-rotor system, or to increase the inter-shaft bearing's radial clearance.

Further studies will be focused on the research of the influence of aerodynamic excitation force on the nonlinear dual-rotor system with multiple defects. The semi-analytical and numerical methods will be used for mutual verification, and the influence of aerodynamic excitation force on the three-rotor system will be investigated. Finally, the experiment will be conducted on the actual machine experimental platform to verify the modelling.

## Data Availability

The datasets used and/or analyzed during the current study available from the corresponding author on reasonable request.
